# Dengue and Chikungunya Seroprevalence in an Urban Low-Income Cohort in Delhi: A Cross-Sectional and Nested Longitudinal Study

**DOI:** 10.3390/tropicalmed11090253

**Published:** 2026-09-09

**Authors:** Nandini Sharma, Saurav Basu, Shivani Rao, Mrunali Zode, Himanshu Bachawandia, Meenakshi Bakshi, Mongjam Meghachandra Singh

**Affiliations:** 1 Department of Community Medicine, Maulana Azad Medical College, New Delhi 110002, India; himanshu41992@gmail.com (H.B.); megharita2@gmail.com (M.M.S.); 2 ESIC Medical College and Hospital, Joka, Kolkata 700104, India; saurav.basu1983@gmail.com; 3 Independent Researcher, New Delhi 122003, India; mzode04@gmail.com; 4 Independent Researcher, New Delhi 122102, India; meenakshi73in@yahoo.com

**Keywords:** dengue, chikungunya, serosurvey, India

## Abstract

**Objectives:** The aim of this study was to ascertain the seroprevalence and determinants of dengue virus (DENV) and chikungunya virus (CHIKV) seropositivity in Delhi, India. **Methods:** This cross-sectional serosurvey with a nested longitudinal immunological sub-study was conducted in an urban resettlement and slum settlement in the North-East district of Delhi in individuals aged ≥2 years. Dengue and Chikungunya virus IgG antibodies were screened using indirect enzyme-linked immunosorbent assay (ELISA), and a longitudinal subset of 100 paired IgG-seropositive samples was further characterized via a plaque reduction neutralization test (µPRNT50) to evaluate changes in functional neutralization over time. **Results:** A total of 752 participants, including 56.65% female participants, were included in the study. Of the participants studied, 81.91% (95% CI: 78.97, 84.60; n = 616) were positive for IgG antibodies against DENV, and 28.32% (95% CI: 25.13, 31.69; n = 213) were positive for CHIKV IgG antibodies. µPRNT50 confirmed stable, near-universal CHIKV neutralization (97% baseline, 98% endline). However, DENV neutralizing antibody showed a significant contraction for DENV-3 (79% baseline, 60% endline) and DENV-4 (69% baseline, 60% endline), while DENV-1 (76%) and DENV-2 (77% to 80%) antibodies remained stable. Individuals living in households with screened windows had significantly lower odds of contracting DENV infection (AOR: 0.36; 95% CI: 0.21, 0.61) and CHIKV infection (AOR: 0.63; 95% CI: 0.43, 0.90) compared with those living in households without screened windows. Further, individuals from households with uncovered containers with stagnant water had 50% higher odds of CHIKV infection than those without such containers (AOR: 1.55; 95% CI: 1.05, 2.29). **Conclusions:** This study revealed a high burden of DENV and CHIKV exposure in this high-risk urban population. While neutralization remained stable for CHIKV, DENV-1, and DENV-2, waning DENV-3 and DENV-4 antibodies suggest differential serotype-specific immunity in the participants.

## 1. Introduction

Arboviruses, including dengue (DENV) and chikungunya (CHIKV), are single-stranded RNA viruses belonging to the Flaviviridae and Togaviridae families, respectively, primarily transmitted through bites of *Aedes aegypti* mosquitoes [1,2,3]. DENV and CHIKV are emerging and remerging infections that are spreading worldwide and thereby representing a major global public health concern, especially in tropical–subtropical countries [4]. DENV has increased 4-fold in the past two decades as per global burden of disease estimates, while both DENV and CHIKV are endemic in more than 100 countries worldwide [5,6].

Despite ongoing vector control programs, mosquito-borne diseases remain a major public health challenge in India. Dengue is hyperendemic across the country, with all four serotypes circulating and frequent outbreaks reported from both urban and rural settings [7,8,9]. A systematic review published in 2018 reported that among 213,285 clinically suspected patients across 180 studies, the pooled prevalence of laboratory-confirmed dengue infection was 38.3% [8]. The same review also reported a pooled dengue seroprevalence of 56.9% based on seven studies. A household-based seroprevalence survey conducted in Chennai reported a very high dengue seropositivity of 93% [10].

Chikungunya re-emerged in India following large-scale outbreaks during 2005–2006, which affected over a million individuals across multiple states, and has since become endemic in several parts of the country [11,12,13,14]. A household-based seroprevalence survey conducted in Chennai reported high CHIKV seropositivity of 44% [10]. Despite its substantial morbidity, particularly chronic arthralgia that may persist for months or even years, chikungunya remains relatively understudied compared to dengue, and population-level data on exposure and associated risk factors remain limited in many regions.

Understanding seroprevalence provides important insights into cumulative population exposure and transmission dynamics, including the burden of asymptomatic and undiagnosed infections. Such evidence is critical for informing surveillance, preparedness, and targeted vector control interventions. While previous studies have documented DENV and CHIKV seroprevalence across various Indian populations, community-based data from high-risk urban settings such as slums and resettlement colonies, particularly the dual burden of these infections, remain limited [8,9]. These settings present unique epidemiological profiles due to the convergence of environmental, behavioural, and socio-demographic risk factors that facilitate vector proliferation and increase human exposure [15].

The present study was therefore conducted to determine the seroprevalence and determinants of DENV and CHIKV seropositivity in an urban resettlement colony and slum in Delhi, India.

## 2. Methods

**Design and Setting:** This was a community-based cross-sectional study conducted as part of a larger multicentric cohort study in an urban resettlement and slum settlement in the North-East district of Delhi, which is also a Demographic Developmental and Environmental Surveillance Site (DDESS) site. The total population of the settlement was 55,000 and was predominantly comprising low-income households. The government health facilities available in the area included an urban primary health facility and an urban Ayushman Arogya Mandir, with both providing outpatient services with limited laboratory services daily. Vector control activities in the area, including fogging and spraying, were predominantly driven by the Municipal Corporation, while information, education, and communication campaigns were periodically conducted by medical students, interns, and frontline health workers.

The area was purposively selected due to its adverse environmental profile with very densely populated clusters, poor sanitation, hygiene, and water scarcity, with multiple dengue and chikungunya outbreaks reported previously. The study was conducted over a period of 6 months (2023–2024).

**Selection criteria:** The study was conducted in individuals above two years of age, and individuals currently residing and likely to stay till the end of one year in the study area. However, those with ongoing fever episodes or a history of acute febrile illness on or within 3 calendar days before enrolment were excluded from the study.

**Sample size and Sampling:** The sample size was calculated for a larger multicentric cohort study with the objective of estimating the incidence of various mosquito-borne diseases, by considering an assumed rate of 5.0 dengue symptomatic cases per 100 years of follow-up, relative precision of 15%, design effect of 2, and accounting for 10% loss to follow-up [16,17,18]. The sample size per site was calculated as 752, which is sufficiently powered for this cross-sectional analysis to estimate the seroprevalence of DENV infection in the study area, considering the expected seroprevalence as 56.9% (based on a systematic review of Indian dengue serosurvey studies), design effect of 2, relative precision of 10%, and an anticipated 10% non-response [8].

To ensure representative age distribution within our sample, we employed age-proportionate stratified sampling. First, a comprehensive household census was utilized to create a line list of all residents within the study site. The population was stratified into the following age groups: 2–4 years (5%), 5–9 years (8%), 10–19 years (18%), 20–49 years (51%), 50–64 years (12%), and 65 years and older (4%), reflecting the community’s age structure. Subsequently, five geographic clusters were created while ensuring each cluster had a population that was four times the sample size to be selected from each cluster. Within each cluster, individuals were randomly selected, maintaining the predetermined age group proportions. This method ensured that our final sample accurately mirrored the age distribution of the study population.

**Procedure:** Trained field investigators visited eligible households for recruitment and data collection from the participants. Among consenting individuals, data were collected through face-to-face interviews with an adult household member present at the time of the visit, preferably the head of the household or another knowledgeable adult household member involved in household decision-making, using a structured participant interview schedule. Subsequently, 5–6 mL of venous blood sample was collected from the participants by a trained phlebotomist under aseptic precautions. All procedures, from collection to storage, were standardized in a comprehensive laboratory manual to maintain strict quality control and an unambiguous chain of custody. Blood samples were collected in the field following good phlebotomy practices and transported to the field lab in vaccine carriers to preserve sample integrity. Upon receipt, meticulous records were logged regarding sample condition, acceptance criteria, and aliquot preparation. Samples were processed within the stipulated timeframe using a calibrated cold centrifuge at 3000 rpm for 15 min. A rigorous barcoding system ensured every aliquot remained fully traceable to its primary sample. Finally, the samples were shipped to NABL-accredited laboratories for ELISA and PRNT testing. Good Clinical Laboratory Practice was followed at the lab level (both field and central) to ensure harmonisation as per assay requirements and as per the highest quality standards.

The samples were screened for both DENV and CHIV IgG antibodies indicative of past exposure to infection. DENV IgG antibody was detected using indirect ELISA (Abbott Diagnostics Korea, Yongin-si, Republic of Korea) with 97.9% sensitivity and 100% specificity. CHIKV IgG antibody was detected using IgG ELISA (InBios International, Seattle, WA, USA) with 90.9% sensitivity and 100% specificity. The assay specific cutoffs defined in the ELISA kit inserts were used to determine antibody positivity. Each batch of testing included quality control and standards to determine batch acceptance.

A subset of 100 paired IgG antibody seropositive samples was randomly selected for confirmatory testing via a micro-plaque reduction neutralization test (uPRNT50) using fluorescence-based microneutralization assays specific to DENV and CHIKV. Neutralizing antibody titres of ≥50 for DENV and ≥10 for CHIKV were established as the thresholds for seropositivity.

Clinical DENV 1-4 isolates, authenticated via whole-genome sequencing, were propagated in C6/36 cells for microneutralization assays (MNA) using LLCMK2 cells. Test sera were heat-inactivated (56 °C, 30 min) and two-fold serially diluted, starting at 1:25. Dilutions were mixed 1:1 with pre-optimized virus stocks (yielding 30–250 foci/well) and incubated at 37 °C for 60 min. The resulting serum-virus complexes were transferred to LLCMK2 monolayers (20,000 cells/well) for a 60 min infection. The inoculum was then replaced with a 2% carboxymethylcellulose overlay. Following a 52 h incubation, cells were fixed with 4% paraformaldehyde, permeabilized, and immunostained using an anti-dengue 2H2 primary antibody and an Alexa 488 secondary antibody. Foci were quantified utilizing a CTL reader equipped with ImmunoSpot 7.0 Pro software. Finally, NT50 neutralization titers were calculated relative to virus controls using SoftMax Pro GxP v7.1.1 software.

**Body Mass Index (BMI):** Weight (kg) and height (cm) were measured during the household visit, and BMI was calculated as weight (kg)/height (m^2^).

**Data and Statistical Analysis:** Data were collected using a paperless data-collection tool on Android-based tablets and subsequently exported to and cleaned in Microsoft Excel. Statistical analyses were performed using Stata version 15.1, StataCorp LLC, College Station, TX.

Descriptive statistics were summarized as frequencies and proportions for categorical variables, and means with standard deviations (SD) for continuous variables. Apparent seroprevalence was estimated, and true seroprevalence was calculated using the Rogan-Gladen estimator, adjusting for assay sensitivity and specificity [15,16,19,20]

*True Prevalence* = *Apparent prevalence* + (*Specificity* − 1)

*Specificity* + (*Sensitivity* − 1)

Univariable logistic regression was performed to assess factors associated with seropositivity, followed by multivariable regression. Selection of variables for the multivariable models was guided by prior literature, biological plausibility, and epidemiological relevance. Key sociodemographic and household environmental variables previously reported to be associated with dengue and chikungunya infection, including age, education, and household environmental conditions, were included in the adjusted models regardless of their significance in univariable analysis [10,21,22,23,24,25]. In addition, variables demonstrating an association with seropositivity in univariable analysis with *p* < 0.05 were included in the multivariable models. Separate models were developed for DENV, CHIKV, and dual seropositivity, and adjusted odds ratios (AORs) with 95% confidence intervals (CIs) were reported. Model assumptions were assessed, and model fit was evaluated using appropriate goodness-of-fit tests. A *p*-value < 0.05 was considered statistically significant.

The proportion of missing data across all variables ranged from 0.13% to 0.93% (occupation: 745/752; caste/tribes: 751/752; animal keeping: 751/752). Given the proportion of missingness was low (<5%), complete case analysis was employed, where participants with missing data for a specific variable were excluded from analyses involving that variable.

## 3. Results

### 3.1. Participant Characteristics

A total of 752 participants were included in the analysis. The mean (SD) age of the participants was 31.46 (17.78) years, including 56.65% female participants. Most participants had completed schooling between years 6 and 10 (34.04%), followed by those with graduate-level education and above (20.88%) and higher secondary schooling (17.29%). A small proportion of participants were illiterate (4.12%). Slightly more than one-third were homemakers, pensioners, senior citizens, or unemployed (37.72%), while 36.24% were students. Around 14% of the participants had a history of at least one chronic medical condition, and 5.85% reported a history of dengue infection (Table 1).

### 3.2. Prevalence of Dengue and Chikungunya Seropositivity

In total, 81.91% (95% CI: 78.97, 84.60; n = 616) of the participants were IgG seropositive for dengue virus. Furthermore, 28.32% (95% CI: 25.13, 31.69; n = 213) of the participants tested positive for chikungunya IgG antibodies. More than one in four participants (26.46%; 95% CI: 23.34, 29.77; n = 199) showed dual positivity indicated by dengue and chikungunya IgG seropositivity.

The true seroprevalence, estimated using the Rogen-Gladen estimator, was 83.51% for dengue, 28.87% for chikungunya, and 26.80% for dengue-chikungunya dual positivity.

### 3.3. Factors Associated with Dengue Seropositivity

**Figure 1:**
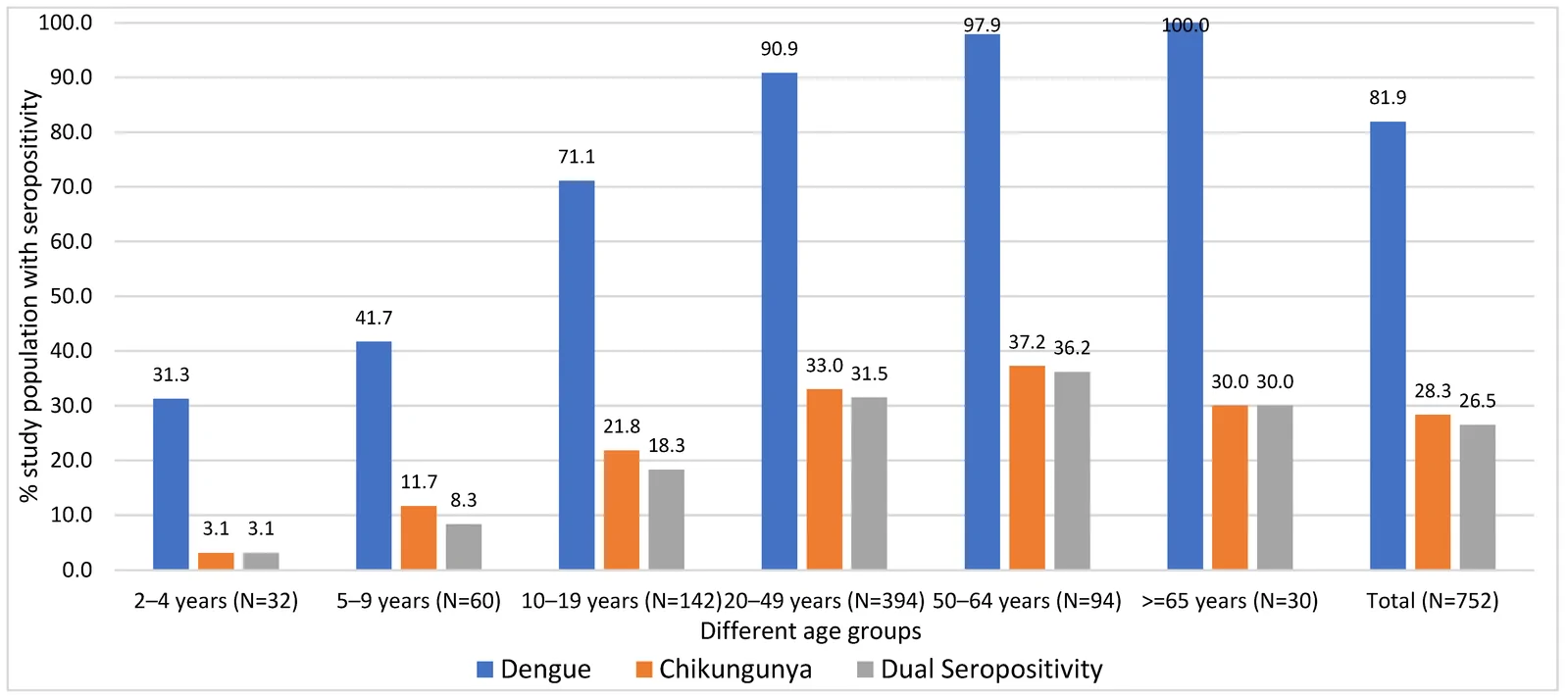
Age-stratified prevalence of DENV, CHIKV, and dual seropositivity (%) (N = 752).

The seroprevalence of dengue virus infection was 48.77% (95% CI: 40.85, 56.73; n = 79/162) among children aged 2–14 years, 89.06% (95% CI: 85.86, 91.74; n = 415/466) in individuals aged 15–49 years, and 98.39% (95% CI: 94.30, 99.80; n = 122/124) among those aged ≥50 years (Figure 1).

Percentages were calculated separately for DENV, CHIKV, and dual seropositivity using the total number of participants in each age category as the denominator.

Increasing age remained strongly associated with dengue seropositivity when adjusted for covariates. Among household-level factors, use of screened windows emerged as an important protective factor and was associated with significantly lower odds of dengue seropositivity (AOR: 0.36; 95% CI: 0.21, 0.61). Other environmental factors, including stagnant water, indoor potted plants, water cooler use, and animal keeping, were not significantly associated after adjustment (Table 2).

### 3.4. Factors Associated with Chikungunya Seropositivity

The seroprevalence of Chikungunya infection was 12.96% (95% CI: 8.21, 19.13; n = 21/162) in children aged 2–14 years, increasing to 31.76% (95% CI: 27.55, 36.20; n = 148/466) among individuals aged 15–49 years, and 35.48% (95% CI: 27.10, 44.58; n = 44/124) in those aged ≥50 years (Figure 1).

Similar to dengue, chikungunya seropositivity increased with age. Environmental conditions appeared to be more strongly associated with risk of chikungunya infection. The presence of uncovered containers with stagnant water was associated with significantly higher odds of chikungunya seropositivity (AOR: 1.55; 95% CI: 1.05, 2.29; *p* = 0.03). Use of screened windows was associated with lower odds of chikungunya seropositivity (AOR: 0.63; 95% CI: 0.43, 0.90; *p* = 0.01) (Table 3).

### 3.5. Factors Associated with Dual Positivity of Dengue and Chikungunya Seropositivity

The seroprevalence of dengue-chikungunya dual positivity was 9.88% (95% CI: 5.75, 15.54; n = 16/162) among children aged 2–14 years, 30.04% (95% CI: 25.91, 34.43; n = 140/466) in individuals aged 15–49 years, and 34.68% (95% CI: 26.36, 43.75; n = 43/124) among those aged ≥50 years (Figure 1).

The age-related pattern observed for dual seropositivity was similar to that seen for both dengue and chikungunya. The presence of uncovered containers with stagnant water was associated with increased odds of dual seropositivity (AOR: 1.67; 95% CI: 1.12, 2.49; *p* = 0.01), while screened windows continued to demonstrate a protective association (AOR: 0.53; 95% CI: 0.36, 0.78; *p* = 0.001) (Table 4).

Overall, age and household environmental conditions were the most consistent factors associated with arboviral seropositivity in this population. While stagnant water increased the risk of chikungunya and dual infection, screened windows consistently showed a protective association with dengue, chikungunya, and dual seropositivity.

To validate the functional immune response within the cohort, 100 individuals who tested positive for IgG via ELISA were further subjected to the gold-standard µPRNT50 assay at both baseline and endline. The results revealed a complex shift in the neutralizing antibody landscape for DENV serotypes. At baseline, neutralizing antibodies were relatively evenly distributed across the four serotypes: DENV-1 (76%), DENV-2 (77%), DENV-3 (79%), and DENV-4 (69%). By the endline, while DENV-1 remained stable (76%) and DENV-2 showed a marginal increase to 80%, there was a notable reduction in neutralization rates for DENV-3 (decreasing to 60%) and DENV-4 (decreasing to 60%). Conversely, Chikungunya (CHIKV) seroprevalence remained near-universal and exceptionally stable throughout the study period, with PRNT positivity recorded at 97% at baseline and 98% at endline.

## 4. Discussion

More than four-fifths of participants were seropositive for dengue, nearly one-third were seropositive for chikungunya, and more than one-fourth showed evidence of exposure to both infections. Together, these findings indicate substantial cumulative exposure to arboviral infections and suggest sustained transmission in an urban resettlement and slum settlement community in Delhi. The observed age-related patterns, environmental conditions, and discordance between ELISA and neutralization findings provide important insights into the epidemiology of DENV and CHIKV and their public health implications in such vulnerable settings.

The high seroprevalence observed in the present study exceeds estimates reported in most previous studies from India, although similarly high seroprevalence has been reported in certain settings. A meta-analysis including seven studies reported a pooled dengue seroprevalence of 56.9% in the general population [8]. Similarly, a multicentre, community-based serosurvey by Murhekar et al. across 15 states reported an overall dengue seroprevalence of 48.7%, with seropositivity increasing with age, a pattern similar to that observed in the current study [26]. The chikungunya seroprevalence of 28.3% observed in our study was also higher than estimates reported from several Indian states [12,27,28]. However, another household-based seroprevalence survey conducted in Chennai reported similarly high seropositivity for both dengue (93%) and chikungunya (44%) [10].

Several factors may have contributed to the elevated seroprevalence in the study population. The study setting, an urban slum and resettlement colony, is characterized by overcrowding, an irregular water supply requiring water storage, poor sanitation, and housing conditions favourable to mosquito breeding and transmission. In addition, recurrent dengue outbreaks and circulation of multiple serotypes in Delhi may contribute to repeated exposure and high cumulative immunity in this population [29,30]. The use of ELISA may detect cross-reactive antibodies from other endemic flaviviruses, potentially contributing to higher estimates [31]. However, the PRNT confirmation in a subset suggests that most ELISA-positive samples likely represent true neutralizing responses.

Household environmental conditions also emerged as important factors linked to arboviral exposure. Uncovered stagnant-water containers were associated with higher odds of chikungunya and dual seropositivity, while screened windows were associated with a protective effect for dengue, chikungunya, and dual seropositivity, highlighting the importance of simple, low-cost household-level vector control measures.

The neutralization findings provided additional insight into the immune landscape of the study population and revealed distinct immunological trajectories for DENV and CHIKV. CHIKV neutralization remained near-universal and stable (97% to 98%), suggesting durable long-term immunity, consistent with the immune response typically observed following alphavirus infections. Similarly, the relative stability observed for DENV-1 and DENV-2 likely reflects recurrent antigenic stimulation from locally circulating strains. In contrast, the significant decline in neutralization for DENV-3 and DENV-4 serotypes points toward the natural longitudinal decay of heterotypic cross-reactive antibodies in the absence of recent exposure. It is important to emphasize that these findings are based on laboratory measures of neutralizing antibodies. Although the observed decline in DENV-3/4 neutralizing titres may indicate reduced serotype-specific humoral immunity at the individual level, the extent to which this translates into increased population susceptibility or outbreak risk cannot be determined from the present study and requires further longitudinal investigation [30].

This study has important public health implications. The high burden of dengue and chikungunya exposure observed in this study suggests growing endemicity of infection in vulnerable Indian urban settings and reinforces the need for strengthened integrated arboviral surveillance, early outbreak detection, and targeted vector control measures. These findings may inform discussions on vaccination strategies in high-burden areas. Public health interventions should continue to prioritize household-level preventive measures, including screened windows and reducing potential mosquito breeding sites through improved sanitation and safe water storage practices.

This study has several strengths, including its community-based design, simultaneous assessment of dengue and chikungunya seroprevalence, and use of both ELISA and µPRNT50 assays to evaluate seropositivity and neutralizing immune responses. Standardized data collection by trained field investigators and laboratory testing in certified laboratories strengthened the validity of the findings. Additionally, the study was conducted in a high-risk urban slum and resettlement colony in Delhi, providing important insights into arboviral burden in a vulnerable setting.

However, the study has some limitations. First, the present findings are from a single site, which may limit generalizability to other settings. Second, the cross-sectional design precluded establishing temporal relationships between exposures and seropositivity outcomes. Third, µPRNT50 analysis was restricted to a randomly selected paired subsample among those with positive IgG seropositivity. Finally, although we adjusted for several sociodemographic and household environmental factors, residual confounding due to unmeasured variables cannot be ruled out.

In conclusion, high seroprevalence, differential serotype-specific immunity, and modifiable household environmental factors in this urban population highlight the complexity of arboviral disease patterns in vulnerable settings. Future multicentric serosurveys incorporating serotype-specific neutralization assays are needed to advance the understanding of arboviral epidemiology and population immunity across diverse settings in the country.

## Figures and Tables

**Table 1:** Sociodemographic characteristics of the study participants.

Characteristics	N = 752
	n (%)
**Age**	
≤14 years	162 (21.54)
15–49 Years	466 (61.97)
≥50 Years	124 (16.49)
**Gender**	
Male	326 (43.35)
Female	426 (56.65)
**Religion**	
Hindu	730 (97.07)
Other	22 (2.93)
**Caste/tribes (N = 751)**	
SC&ST	418 (55.66)
OBC	149 (19.84)
Others	184 (24.50)
**Marital status**	
Married	391 (51.99)
Unmarried/Divorced/Widowed	361 (48.01)
**Education level**	
Illiterate	31 (4.12)
Can read or sign/Anganwadi/Preschool	75 (9.97)
School year 1 to 5	103 (13.7)
School year 6 to 10	256 (34.04)
Higher secondary schooling	130 (17.29)
Graduate and above	157 (20.88)
**Occupation** (N = 745)	
Business/Trader/Shopkeeper/Self-employed	53 (7.11)
Government or Private job	109 (14.63)
Skilled Labour/menial workers	32 (4.3)
Homemakers/Pensioners/Senior citizens/Unemployed	281 (37.72)
Student	270 (36.24)
**History of chronic illnesses**	
Yes	103 (13.70)
**BMI (kg/m^2^)**	22.45 (5.60)
**History of dengue infection**	
Yes	44 (5.85%)

SC: Scheduled Castes, ST: Scheduled Tribes, OBC: Other Backward Classes; Analyses were based on available data for each variable; denominators vary accordingly.

**Table 2:** Distribution of factors associated with Dengue seropositivity.

Factors	Total	Dengue Seropositive	Univariable Analysis		Multivariable Analysis	
		n (%)	COR (95% CI)	*p* Value	AOR (95% CI)	*p* Value
	N = 752	n = 616				
**Age, mean (SD)**	31.46 (17.78)	34.83 (16.86)	1.09 (1.07, 1.11)	<0.001	1.08 (1.05, 1.12)	<0.001
**Gender**						
Male	326	262 (42.53)	1		1	
Female	426	354 (57.47)	1.20 (0.83, 1.74)	0.003	0.87 (0.52, 1.48)	0.62
**Caste/tribes**	N = 751					
SC&ST	418	352 (57.24)	1.94 (1.27, 2.95)	0.002	1.81 (1.07, 3.08)	0.03
OBC	149	128 (20.81)	2.21 (1.26, 3.89)	0.006	2.56 (1.29, 5.1)	0.007
Others	184	135 (21.95)	1		1	
**Education level**						
Illiterate	31	30 (4.87)	1		1	
Can read or sign/Anganwadi/Preschool	75	46 (7.47)	0.05 (0.01,0.41)	0.005	0.65 (0.07, 6.24)	0.71
School year 1 to 5	103	62 (10.06)	0.05 (0.01, 0.38)	0.004	0.74 (0.08, 6.79)	0.78
School year 6 to 10	256	221 (35.88)	0.21 (0.03, 1.59)	0.131	1.84 (0.21, 16.01)	0.58
Higher secondary schooling	130	116 (18.83)	0.28 (0.03, 2.18)	0.223	2.86 (0.31, 26.14)	0.35
Graduate and above	157	141 (22.89)	0.29 (0.04, 2.3)	0.243	2.12 (0.24, 18.67)	0.50
**Occupation**	N = 745	n = 609				
Business/TraderShopkeeper/Self-employed	53	52 (8.54)	1		1	
Government or Private job	109	100 (16.42)	0.21 (0.03, 1.73)	0.148	0.22 (0.03, 1.89)	0.17
Skilled Labour/menial workers	32	31 (5.09)	0.6 (0.04, 9.87)	0.718	0.46 (0.03, 8.28)	0.60
Homemakers/Pensioners/Senior citizens/Unemployed	281	259 (42.53)	0.23 (0.03, 1.72)	0.151	0.22 (0.03, 1.77)	0.15
Student	270	167 (27.42)	0.03 (0.004,0.23)	0.001	0.21 (0.02, 1.8)	0.16
**History of chronic illnesses**						
Yes	103	97 (15.75)	4.05 (1.74, 9.44)	0.001	1.00 (0.37, 2.69)	0.99
No	649	519 (84.25)	1		1	
**BMI (kg/m^2^), mean (SD)**	22.45 (5.60)	23.25 (5.28)	1.18 (1.13, 1.23)	<0.001	0.97 (0.91, 1.03)	0.33
**The presence of uncovered containers with stagnant water, whether indoors or outdoors**						
Yes	211	182 (29.55)	1.55 (0.99, 2.42)	0.055	1.26 (0.71, 2.26)	0.43
No	541	434 (70.45)	1		1	
**Presence of indoor potted plants**						
Yes	472	390 (63.31)	1.14 (0.78, 1.66)	0.510	0.95 (0.59, 1.53)	0.83
No	280	226 (36.69)	1		1	
**Use of water cooler**						
Yes	671	551 (89.45)	1.13 (0.63, 2.02)	0.680	1.11 (0.53, 2.32)	0.79
No	81	65 (10.55)	1		1	
**Animal keeping in house/neighbourhood**	N = 751	n = 615				
Yes	81	69 (11.22)	1.31 (0.69, 2.49)	0.416	0.82 (0.38, 1.78)	0.61
No	670	546 (88.78)	1		1	
**Use screened windows**						
Yes	392	308 (50.00)	0.62 (0.42, 0.91)	0.013	0.36 (0.21, 0.61)	<0.001
No	360	308 (50.00)	1		1	
**History of the previous dengue infection**						
Yes	44	36 (5.84)	0.99 (0.45, 2.19)	0.986	-	
No	708	580 (94.16)	1			

Estat gof *p* value = 0.14 (*p*-value from the Hosmer–Lemeshow goodness-of-fit test for the final multivariable logistic regression model); N = 743; SC: Scheduled Castes, ST: Scheduled Tribes, OBC: Other Backward Classes; Analyses were based on available data for each variable; denominators vary accordingly.

**Table 3:** Distribution of factors associated with Chikungunya seropositivity.

Factors	Total	Chikungunya Seropositive	Univariable Analysis		Multivariable Analysis	
		n (%)	COR (95% CI)	*p* Value	AOR (95% CI)	*p* Value
	N = 752	n = 213				
**Age, mean (SD)**	31.46 (17.78)	35.96 (16.13)	1.02 (1.01, 1.03)	<0.001	1.02 (1.01, 1.03)	0.001
**Gender**						
Male	326	99 (46.48)	1		-	
Female	426	114 (53.52)	0.84 (0.61, 1.15)	0.277		
**Caste/tribes**						
SC&ST	418	119 (55.87)	1.16 (0.78, 1.72)	0.460	-	
OBC	149	47 (22.07)	1.34 (0.83, 2.17)	0.227		
Others	184	47 (22.07)	1			
**Education level**						
Illiterate	31	11 (5.16)	1		1	
Can read or sign/Anganwadi/Preschool	75	16 (7.51)	0.49 (0.20, 1.24)	0.132	0.97 (0.36, 2.6)	0.95
School year 1 to 5	103	16 (7.51)	0.33 (0.13, 0.83)	0.018	0.85 (0.31, 2.32)	0.75
School year 6 to 10	256	74 (34.74)	0.74 (0.34, 1.62)	0.45	1.46 (0.63, 3.39)	0.38
Higher secondary schooling	130	36 (16.90)	0.70 (0.30, 1.60)	0.393	1.6 (0.64, 3.97)	0.31
Graduate and above	157	60 (28.17)	1.12 (0.50, 2.51)	0.774	2.62 (1.09, 6.31)	0.03
**Occupation**	N = 745	n = 212				
Business/Trader/Shopkeeper/Self-employed	53	15 (7.08)	1		-	
Government or Private job	109	47 (22.17)	1.92 (0.95, 3.90)	0.071		
Skilled Labour/menial workers	32	14 (6.60)	1.97 (0.79, 4.94)	0.148		
Homemakers/Pensioners/Senior citizens/Unemployed	281	87 (41.04)	1.14 (0.59, 2.17)	0.700		
Student	270	49 (23.11)	0.56 (0.29, 1.10)	0.093		
**History of chronic illnesses**						
Yes	103	36 (16.90)	1.43 (0.92, 2.23)	0.109	-	
No	649	177 (83.10)	1			
**BMI (kg/m^2^), mean (SD)**	22.45 (5.60)	23.37 (5.40)	1.04 (1.01, 1.07)	0.005	1.00 (0.96, 1.04)	0.96
**The presence of uncovered containers with stagnant water, whether indoors or outdoors**						
Yes	211	73 (34.27)	1.52 (1.08, 2.13)	0.017	1.55 (1.05, 2.29)	0.03
No	541	140 (65.73)			1	
**Presence of indoor potted plants**						
Yes	472	134 (62.91)	1.00 (0.73, 1.40)	0.959	0.87 (0.61, 1.24)	0.43
No	280	79 (37.09)	1		1	
**Use of water cooler**						
Yes	671	188 (88.26)	0.87 (0.53, 1.44)	0.591	0.86 (0.51, 1.47)	0.59
No	81	25 (11.74)	1		1	
**Animal keeping in house/neighbourhood**	N = 751					
Yes	81	26 (12.21)	1.22 (0.74, 2.00)	0.430	1.20 (0.71, 2.03)	0.50
No	670	187 (87.79)	1		1	
**Use screened windows**						
Yes	392	96 (45.07)	0.67 (0.49, 0.93)	0.015	0.63 (0.43, 0.90)	0.01
No	360	117 (54.93)	1		1	

Estat gof *p* value = 0.47 (*p*-value from the Hosmer–Lemeshow goodness-of-fit test for the final multivariable logistic regression model); N = 751; SC: Scheduled Castes, ST: Scheduled Tribes, OBC: Other Backward Classes; Analyses were based on available data for each variable; denominators vary accordingly.

**Table 4:** Distribution of factors associated with dual positivity of Dengue and Chikungunya.

Factors	Total	Dual Positivity	Univariable Analysis		Multivariable Analysis	
		n (%)	COR (95% CI)	*p* Value	AOR (95% CI)	*p* Value
	N = 752	n = 199				
**Age, mean (SD)**	31.46 (17.78)	36.58 (15.87)	1.02 (1.01, 1.03)	<0.001	1.02 (1.01, 1.04)	<0.001
**Gender**						
Male	326	93 (46.73)	1		-	
Female	426	106 (53.27)	0.83 (0.60, 1.15)	0.262		
**Caste/tribes**						
SC&ST	418	113 (56.78)	1.21 (0.81, 1.82)	0.345	-	
OBC	149	43 (21.61)	1.33 (0,81, 2.18)	0.256		
Others	184	43 (21.61)	1			
**Education level**						
Illiterate/Can read or sign/Anganwadi/Preschool	106	26 (13.07)	1		1	
School year 1 to 10	359	81 (40.70)	0.90 (0.54, 1.49)	0.673	1.31 (0.75, 2.28)	0.34
Higher secondary schooling	130	34 (17.09)	1.09 (0.60, 1.97)	0.775	1.81 (0.94, 3.49)	0.08
Graduate and above	157	58 (29.15)	1.80 (1.04, 3.12)	0.035	3.02 (1.62, 5.63)	0.001
**Occupation**	N = 745	n = 198				
Business/TraderShopkeeper/Self-employed	53	14 (7.07)	1		-	
Government or Private job	109	45 (22.73)	1.96 (0.95, 4.02)	0.067		
Skilled Labour/menial workers	32	14 (7.07)	2.17 (0.86, 5.48)	0.102		
Homemakers/Pensioners/Senior citizens/Unemployed	281	83 (41.92)	1.17 (0.60, 2.26)	0.646		
Student	270	42 (21.21)	0.51 (0.26, 1.03)	0.059		
**History of chronic illnesses**						
Yes	103	33 (16.58)	1.37 (0.87, 2.15)	0.169	-	
No	649	166 (83.42)	1			
**BMI (kg/m^2^), mean (SD)**	22.45 (5.60)	23.50 (5.25)	1.05 (1.02, 1.08)	0.002	1.01 (0.97, 1.05)	0.74
**Presence of uncovered containers with stagnant water**						
Yes	211	71 (35.68)	1.64 (1.16, 2.32)	0.005	1.67 (1.12, 2.49)	0.01
No	541	128 (64.32)	1		1	
**Presence of indoor potted plants**						
Yes	472	125 (62.81)	1.00 (0.72, 1.40)	0.987	0.85 (0.59, 1.21)	0.36
No	280	74 (37.19)	1		1	
**Use of water cooler**						
Yes	671	176 (88.44)	0.90 (0.54, 1.50)	0.677	0.85 (0.49, 1.47)	0.55
No	81	23 (11.56)	1			
**Animal rearing in house/neighbourhood**	N = 751					
Yes	81	23 (11.56)	1.11 (0.67, 1.86)	0.682	1.08 (0.62, 1.86)	0.79
No	670	176 (88.44)	1		1	
**Use screened windows**						
Yes	392	85 (42.71)	0.60 (0.43, 0.83)	0.002	0.53 (0.36, 0.78)	0.001
No	360	114 (57.29)	1		1	
**History of Dengue infection**						
Yes	44	13 (6.53)	1.18 (0.60, 2.30)	0.633	-	
No	708	186 (93.47)	1			

Estat gof *p* value = 0.62 (*p*-value from the Hosmer–Lemeshow goodness-of-fit test for the final multivariable logistic regression model); N = 751; SC: Scheduled Castes, ST: Scheduled Tribes, OBC: Other Backward Classes; Analyses were based on available data for each variable; denominators vary accordingly.

## Data Availability

Data that support the findings of this study will be made available from the corresponding author upon reasonable request.
